# Influence of Mediterranean Diet on Blood Pressure

**DOI:** 10.3390/nu10111700

**Published:** 2018-11-07

**Authors:** Giovanni De Pergola, Annunziata D’Alessandro

**Affiliations:** 1 Clinical Nutrition Unit, Medical Oncology, Department of Biomedical Science and Human Oncology, School of Medicine, University of Bari, Policlinico, Piazza Giulio Cesare 11, 70124 Bari, Italy; 2 General Medicine A.S.L. Bari, 70126 Bari, Italy; a.dalessandro2011@libero.it

**Keywords:** Mediterranean Diet, blood pressure

## Abstract

Hypertension is the main risk factor for cardiovascular disease (CVD) and all-cause mortality. Some studies have reported that food typical of the Mediterranean diet (MedDiet), such as whole grains, vegetables, fruits, nuts, and extra virgin olive oil, have a favorable effect on the risk of hypertension, whereas food not typical of this dietary pattern such as red meat, processed meat, and poultry has an unfavorable effect. In this review, we have summarized observational and intervention studies, meta-analyses, and systematic reviews that have evaluated the effects of the MedDiet as a pattern towards blood pressure (BP). However, the number of such studies is small. In general terms, the MedDiet has a favorable effect in reducing BP in hypertensive or healthy people but we do not have enough data to declare how strong this effect is. Many more studies are required to fully understand the BP changes induced by the MedDiet.

## 1. Introduction

Hypertension is the main risk factor for cardiovascular diseases (CVD) and all-cause mortality [1]. A healthful lifestyle is a fundamental strategy for decreasing hypertension, and diet is the changeable element with the strongest effect on blood pressure (BP) [2]; there is evidence that the pattern of the Mediterranean diet (MedDiet) may improve endothelial function [3] and offer a considerable benefit against the risk of hypertension and CVD [3,4,5]. 

The main components of the MedDiet are vegetables, fresh fruit, whole grains, fish and seafood, legumes, nuts, extra virgin olive oil, and red wine, whereas red and processed meat are limited, and dairy foods are moderate [6,7,8]. 

In fact, there are not many studies that explore the influence of the MedDiet on BP, and the available studies have not obtained results that establish an agreement on the effect of the MedDiet in the prevention and care of hypertension. This may be due to several reasons: (1) the MedDiet has some differences according to the geographical area; (2) the age under observation was different throughout the studies; (3) observational or intervention studies have been performed to evaluate the relationship between the MedDiet and hypertension; (4) blood pressure was monitored at home (or in the office) in most of the studies, whereas the more reliable 24-h ambulatory blood pressure measurement (ABPM) was used only in one study; (5) some studies examined normotensive subjects whereas others examined hypertensive patients; (6) some studies lasted for less than one year, whereas others were performed for more than four years; and (7) the control groups were characterized differently throughout the studies. Lastly, we should not forget that the MedDiet described in the studies commonly examined in the meta-analyses has quantitative and/or qualitative differences compared to the traditional MedDiet of the early 1960s [9,10]. However, a positive aspect of almost all the available studies on the relationship between the MedDiet and BP is that adherence to the MedDiet is highly significant since this diet is palatable and satiating [6,7,8]. 

## 2. The MedDiet and Blood Pressure: Observational Studies 

The Greek European Prospective Investigation into Cancer and Nutrition (EPIC) study examined 20,343 participants who did not have a diagnosis of hypertension. It demonstrated that the MedDiet score was significantly and negatively associated with both systolic (SBP) and diastolic blood pressure (DBP) [11]. The study by the Seguimiento University of Navarra (SUN), a Spanish prospective cohort study, investigated the relationship between adherence to the MedDiet and the incidence of hypertension in a population of 9408 men and women [12]. The participants were all university graduates, nurses, and other educated adults. Adherence to the MedDiet was related to small changes in mean levels of SBP and DBP after six years of follow-up, suggesting that adhering to a MedDiet could contribute to preventing changes in BP related to age [12]. The most recent work on this topic is the study by the Florence cohort of the cross-sectional EPIC [13]. This study shows that the Italian Mediterranean Index was significantly and negatively correlated with SBP and DBP values in a total population of 13,597 volunteers (aged 35–64 years) enrolled in the period from 1993 to 1998. At variance with the Italian score, the Greek MedDiet score was not associated with SBP and DBP. It is possible that the use of tertiles of food intake, as in the Italian Mediterranean Index, provides a better classification than the use of the median of food intake as in the Greek MedDiet score for the adherence to a healthy diet.

The ATTICA study is a population-based cohort randomly enrolling 3042 adults belonging to the greater area of Athens. In one of the works related to this study, the authors studied only participants with an excess body weight, and the multivariate analysis demonstrated that SBP was independently and negatively, but only modestly, associated to the MedDiet [14]. Therefore, this is the only study examining adherence to the MedDiet that did not show a protective effect of the MedDiet on DBP. This is possibly explained by the fact that only overweight and obese subjects were examined, and it is well-known that obesity has its own hormone and hemodynamic characteristics [15]. 

## 3. The MedDiet and Blood Pressure: Intervention Studies 

Results from randomized and controlled trials (RTCs) performed with dietary interventions are more relevant since they have the highest potential to influence dietary guidelines, practices and healthcare policies with the main aim of improving public health. Thus, if we take into account the intervention studies, the Prevención con Dieta Mediterránea (PREDIMED) study was performed in two Spanish centers involving >7000 subjects with the complex end point of myocardial infarction, stroke and cardiovascular death as the primary outcome. In particular, this study compared the MedDiet with a low-fat control diet in 7447 men (aged 55 to 80 years) and women (aged 60 to 80 years), and more than 80% of these subjects had hypertension [16]. After a follow-up period of 4 years, the PREDIMED study showed no change in SBP in both groups, whereas DBP was decreased by 1.5 and 0.7 mm Hg in the extra virgin olive oil and in the mixed nuts MedDiet intervention groups, respectively [16]. 

Davis et al. recently performed an RTC to examine the influence of an increased adherence to a MedDiet for 6 months on BP in Australian subjects represented by 166 healthy men and women, aged > 64 years [17]. This study showed that Australian subjects who consumed a MedDiet for 6 months had a small but significantly lower SBP after either 3 or 6 months as compared to subjects who maintained their habitual diet, and improved endothelial function [17]. 

All the above studies were performed using home or office BP measurements. This approach has limitations because of poor reproducibility, observer and patient variability, and white-coat effect [18]. By contrast, 24-h ABPM is the gold standard for examining the influence of different interventions on BP, because repeated measurements reflect usual BP more accurately than single office measurements [19]. 

The only study performed using 24-h ABPM to evaluate the control of BP under the MedDiet is the PREDIMED study performed by Doménech et al. [20], who reported results from a dietary intervention with 3 arms in subjects mostly affected by hypertension (85%). The 1-year trial consisted of a MedDiet supplemented with either extra virgin olive oil or mixed nuts that was compared with a control diet in which participants had to reduce their dietary fat intake. The participants were 235 women and men, aged from 55 to 80 years. After 1 year, the extra virgin olive oil and mixed nuts groups had, respectively, 4.0 and 4.3 mm Hg lower mean SBP 24-h and 1.9 and 1.9 mm Hg lower mean DBP 24-h than the control diet group [20].

Furthermore, dietary intervention trials may have some limitations. First, they cannot be evaluated in a double blind, placebo-controlled way and may suffer from non-adherence, crossover between studied diets, and lack of blinding. Second, the participants should maintain their body weight and the different kinds of treatment should be isocaloric, and this is not an easy task to accomplish. Third, dietary interventions often require a long period to give results and may therefore suffer from an overly short duration. Further disadvantages may include the fact that a change in consumption of one food often modifies the consumption of other foods. Theoretically, meta-analyses and systematic reviews may provide more information.

## 4. Meta-analyses and Systematic Reviews

Two meta-analyses, published in 2016, gave opposite conclusions. The first meta-analysis included RTCs lasting at least 12 months, with a low-fat control group. Although both SBP and DBP showed a significant reduction, the authors declared themselves to be unconvinced that the MedDiet lowers BP more than the low-fat diet, suggesting that the doubt result was possibly due to the limited number and heterogeneity of the studies (*n* = 7) [21]. Another meta-analysis of RTCs showed that the MedDiet lowers SBP by 3.02 mm Hg and DBP by 1.99 mm Hg [22]. Only five MedDiet studies were included for the statistical analysis: Two of these studies did not identify significant effects on BP, but were excluded from the analysis because the data were incomplete. Moreover, both meta-analyses considered data from the PREDIMED study, but the first meta-analysis, giving a negative conclusion, used the data from the 24-month follow-up, whereas the second, which gave a positive conclusion, used the findings from the 12-month follow-up. 

## 5. Specific Food Typical of the MedDiet that Influences Blood Pressure

Most of the influence of the MedDiet is mediated by the combined effects of complete dietary habits; however, some specific foods might be more effective than others. Olive oil is possibly the most important component of the MedDiet from this point of view. The mutual adjustment of data in the Greek EPIC study showed that olive oil has the most favorable effect on BP in this population [11]. Interestingly, recent studies have reported a vasoprotective effect of polyphenols present in olive oil on blood pressure and explained this effect by the power to increase the endothelial synthesis of nitric oxide and the response mediated by the endothelium-derived hyperpolarization factor [23,24]. Apart from olive oil, dietary intakes of fruit and vegetables, nuts and whole grain have been related to a lower risk of hypertension [25]. 

## 6. Influence of Sodium and Potassium Intake

The simultaneous influence of sodium and potassium intake should be taken into account when the effect of the MedDiet on BP is examined. In fact, a recent study showed that a higher adherence to the MedDiet was negatively related to hypertension, but this association was no more significant after adjustment for sodium and potassium intake [26].

## 7. Comparison with Other Healthy Diets

Concerning the type of diet and BP, the Dietary Approaches to Stop Hypertension (DASH) diet was the first dietary approach reporting to show a clear effect in reducing BP in subjects with BP ≥ 120/80 mmHg [27,28]. It should be noted that a recent systematic review and network meta-analysis of RTCs compared the effects of 13 different dietary proposals (Mediterranean, DASH, low-fat, moderate-carbohydrate, high-protein, low-carbohydrate, Palaeolithic, vegetarian, low-GI/GL, low-sodium, Nordic, Tibetan, and control) on blood pressure in pre-hypertensive and hypertensive patients [29], demonstrating that the DASH diet is the most effective dietetic measure to reduce BP. The authors did not explain their results, but it would be very interesting to understand the explanation for this finding. Both the MedDiet and the DASH diet are relatively easy to adhere to and are palatable, high in fruit, vegetables, whole grains, nuts, and unsaturated oils [30]; moreover, both minimize the consumption of red and processed meat, and are in accordance with dietary recommendations for cardiovascular health. Thus, what are the differences? One may be that the DASH diet is more suitable for recommending a low sodium intake [27,28], whereas this is not a feature of the MedDiet. Second, it may well be that the DASH diet includes more proteins since it includes poultry and fish and emphasizes the consumption of free- or low-fat dairy products (two or three servings per day) [27,28]. In this regard, either a higher protein intake or protein supplementation have been shown to decrease blood pressure [31,32]. Concerning dairy products in particular, the addition of conventional non-fat dairy products to the routine diet has hypotensive effects [33]. Moreover, a recent systematic review has shown a favorable association between a higher dairy intake and a lower risk of hypertension [34].

## 8. Conclusions

The MedDiet is undoubtedly a healthy diet model, which is effective in protecting against CVD, metabolic diseases, and cancer. Some studies have reported that foods typical of the MedDiet of the early 1960s, such as whole grains, vegetables, fruit, nuts, and extra virgin olive oil, have a favorable effect in the risk of hypertension [25,35,36,37] whereas foods not typical of this dietary pattern, such as red meat, processed meat, and poultry, have an unfavorable effect [25,38]. A few studies have evaluated the effect of the MedDiet as a pattern towards BP. In general terms, current studies indicate that the MedDiet has favorable effects in reducing BP in hypertensive or healthy people but we do not have enough data to declare how strong this effect is. Seemingly, we do not have data about the effects of the MedDiet in the presence of specific diseases (diabetes, etc.). We are convinced that far more studies are required to understand the BP changes induced by the MedDiet.
